# Severe Ascites in Common Variable Immunodeficiency

**DOI:** 10.7759/cureus.30274

**Published:** 2022-10-13

**Authors:** Guilherme Camões, Diogo A Fernandes, Diana M Ferreira, Arsénio Santos, Armando Carvalho

**Affiliations:** 1 Internal Medicine, Centro Hospitalar e Universitário de Coimbra, Coimbra, PRT; 2 Cardiology, Centro Hospitalar e Universitário de Coimbra, Coimbra, PRT

**Keywords:** hepatology, nodular regenerative hyperplasia, ascites, idiopathic non-cirrhotic portal hypertension, common variable immunodeficiency

## Abstract

Liver disease is one of the possible clinical manifestations of common variable immunodeficiency and can range from mild hepatomegaly and persistent elevation of liver enzymes to cirrhosis, portal hypertension, and nodular regenerative hyperplasia. The last one is the most common histologic presentation of liver involvement by common variable immunodeficiency and its clinical spectrum can range from asymptomatic to cholestasis, liver cirrhosis, or idiopathic non-cirrhotic portal hypertension, with the severe manifestations being less recognised. We present a case of a 48-year-old woman who was referred for an internal medicine consultation for evaluation of rapidly progressing (span of three months) large-volume ascites and marked asthenia. The patient had a past medical history of common variable immunodeficiency and a recent episode of severe haemolytic anaemia. Peritoneal fluid analyses identified portal hypertension as the cause of the ascites. Abdominal Doppler ultrasound and contrasted abdominal computed tomography confirmed the presence of permeable hepatic and portal veins. Liver biopsy revealed regenerative nodular hyperplasia without cirrhosis. A diagnosis of idiopathic non-cirrhotic portal hypertension secondary to common variable immunodeficiency was made. Treatment was adjusted with considerable improvement in ascites. In conclusion, idiopathic non-cirrhotic portal hypertension is a possible and often overlooked complication in patients with common variable immunodeficiency and is an exclusion diagnosis that requires a high level of suspicion, especially in patients with ascites.

## Introduction

Common variable immunodeficiency (CVID) is one of the most common primary immunodeficiencies and results from a failure of B lymphocyte differentiation associated with a decrease in antibody production and consequent hypogammaglobulinemia [1-5]. Patients with CVID will have a wide array of clinical manifestations, such as recurrent infections, chronic pulmonary disease, autoimmune diseases, granulomatosis diseases, gastrointestinal diseases, liver disease, and neoplasms [3-5]. Liver disease is estimated to be present in about 10% of patients and is often overlooked [4,6].

Liver involvement in CVID ranges from mild hepatomegaly and persistent elevation of liver enzymes (most commonly alkaline phosphatase) in nearly 50% of patients to cirrhosis, portal hypertension (PH), and nodular regenerative hyperplasia (NRH) [1-6]. NRH is the main cause of idiopathic non-cirrhotic PH (INCPH) in western countries, accounting for 27% of the cases in Europe [1]. It is regarded as the most common histologic presentation of liver involvement by CVID with a prevalence of about 5% [2,3,6,7]. Its clinical spectrum can range from asymptomatic to cholestasis, liver cirrhosis [3,4], or INCPH [8,9], a rare disease that manifests through PH [9]. With this case report, we highlight the INPCH as a rare presentation of CVID.

## Case presentation

A 48-old woman, previously diagnosed with CVID, was referred to Internal Medicine for marked asthenia for small efforts and rapidly progressing large abdominal distension (three months). The patient also presented with dyspnoea, xerophthalmia, and xerostomia.

The patient’s medical history included typhoid fever at 11 years of age, mononucleosis when she was 13 years, sarcoidosis (pulmonary granulomas) at 34 years of age, and CVID (multiple episodes of severe pneumonia with empyema) at 45 years of age. Three months before the new symptoms, she was admitted with severe haemolytic anaemia, a complication of CVID, requiring high doses of corticosteroids and rituximab for adequate control.

Her home medications included escitalopram 10mg, pantoprazole 20mg, drospirenone 3mg/ethinylestradiol 0.03mg, and IgG human immunoglobulin (25g monthly dose subcutaneous). After the last hospitalization, caused by severe haemolytic anaemia, she started prednisolone 15mg, spironolactone 150mg, and furosemide 40mg twice a day. She denied any consumption of drugs, toxins, alcohol, herbal products, and tobacco. The patient denied any relevant family history.

Physical examination revealed normal blood pressure, tachycardia (111 bpm), abdominal perimeter of 94cm, height of 1.69m, weight of 70kg, discoloured mucous membranes, sarcopenia, and apparent dyspnoea with mild exertion. Cardiac and pulmonary auscultation was normal. The abdomen was severely distended, impossible to palpate organomegalies, with tense but painless ascites and pronounced collateral venous circulation. The fluid wave test for ascites was positive. No oedema in the lower limbs. The remaining physical examination provided no additional relevant information.

Investigations

Due to the recent complication of CVID with severe haemolytic anaemia, the patient had already undergone extensive investigation. From this, the following results stood out: macrocytic anaemia, increased reticulocyte production, leukopenia with lymphopenia, unconjugated hyperbilirubinemia, and increased lactate dehydrogenase, without changes in liver enzymes or changes in coagulation. More details are given in Table 1.

**Table 1:** Laboratory results

	Patient value	Reference value
Haemoglobin	10.2 g/dL	12-16
Hematocrit	29.5%	36-46
Mean globular volume	113 fL	80-100
Reticulocytes (%)	19%	1-2%
Reticulocytes (N)	497.7x10^9^/L	50-100
Erythrocyte production index	6,25	
Leukocytes	2.97x10^3^/µL	4-10
Neutrophils	2.28x10^3^/µL	2-7
Lymphocytes	0.4x10^3^/µL	1-3
Platelets	189x10^3^/µL	150-400
Creatine	0.81 mg/dL	0.52-1.04
Urea nitrogen	52 mg/dL	15-38
Albumin	38 g/dL	35-50
Aspartate transaminase	24 U/L	<31
Alanine transaminase	21 U/L	<34
Alkaline phosphatase	78 U/L	30-120
Gamma-glutamyl transferase	30 U/L	<38
Total bilirubin	2.30 mg/dL	0.3-1.2
Direct bilirubin	0.88 mg/dL	0.1-0.5
Lactate dehydrogenase	438 U/L	125-220
Prothrombin time	10.3s	9.4-12.5
Activated partial thromboplastin time	24.5s	25-34
International normalized ratio	0.91	

Urinalysis, myelogram with bone biopsy, and echocardiogram were normal. Diagnostic paracentesis was performed and the study of ascitic fluid revealed a sero-ascitic gradient of albumin of 2.4g/dL (compatible with PH) and absence of neoplastic cells and infection of the peritoneal fluid.

To ascertain the aetiology of the PH, multiple studies were performed that led to the exclusion of several diseases, such as diseases of the red blood cell, infectious diseases that fit the patient's presentation and their risk factors (including viral hepatitis), autoimmune diseases, alpha-1-antitrypsin deficiency, hemochromatosis, celiac disease, inflammatory bowel disease, and Wilson's disease. We also performed a deep analysis of the patient's clinical history and it was evident that the patient did not have, nor had she ever had, sarcoidosis.

**Figure 1:**
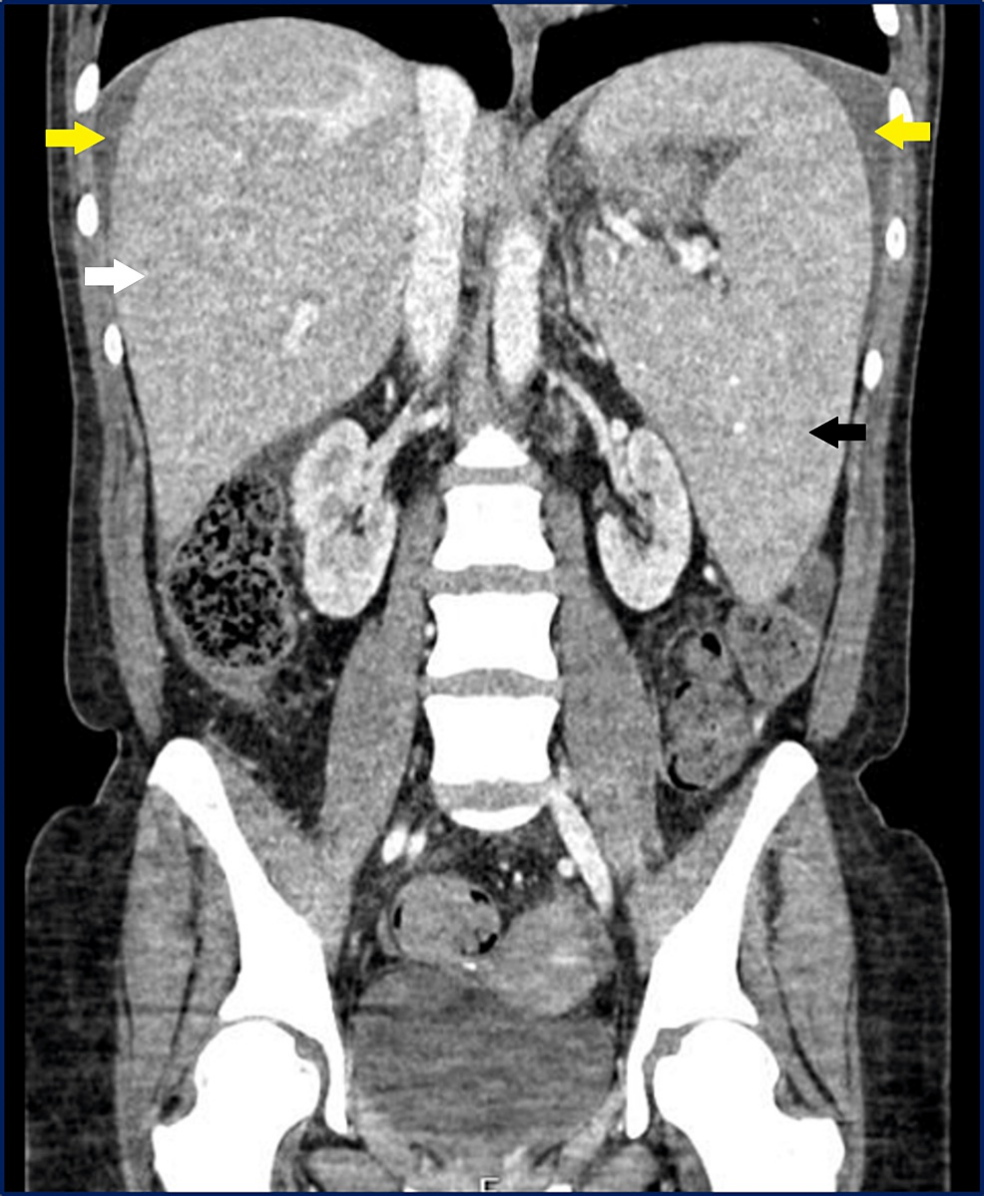
Coronal section of abdominal computed tomography showing hepatomegaly (white arrow), splenomegaly (black arrow), and free peritoneal effusion (yellow arrow).

**Figure 2:**
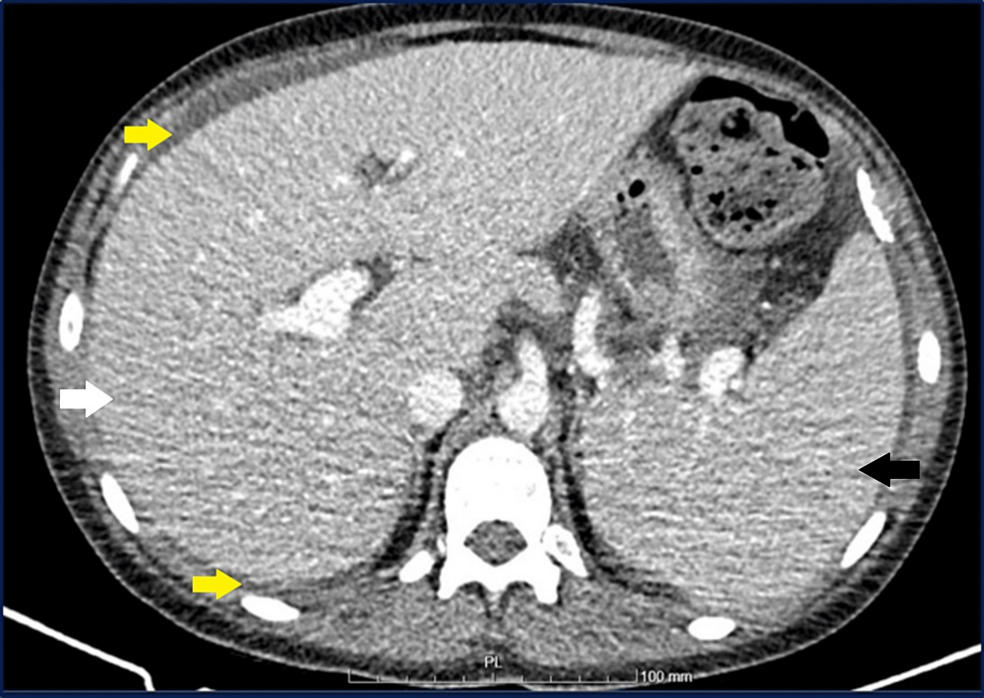
Cross-section of abdominal computed tomography showing hepatomegaly (white arrow), splenomegaly (black arrow), and free peritoneal effusion (yellow arrow).

Abdominal ultrasound and chest and abdominal computerized tomography revealed hepatomegaly (19.8cm), splenomegaly (19.5cm), and large free peritoneal fluid (Figures 1, 2). Abdominal Doppler ultrasound revealed a permeable portal vein with calibre at the upper limit of normality (13mm), with hepatopetal flow and normal velocities (26cm/s), a hepatic artery without changes, and hepatic veins with hepatofugal flow and reduced cardiac and respiratory modulation. Also, the abdominal computerized tomography with contrast excludes portal and hepatic vein thrombosis. Both exams excluded pre- and post-hepatic thrombotic causes.

**Figure 3:**
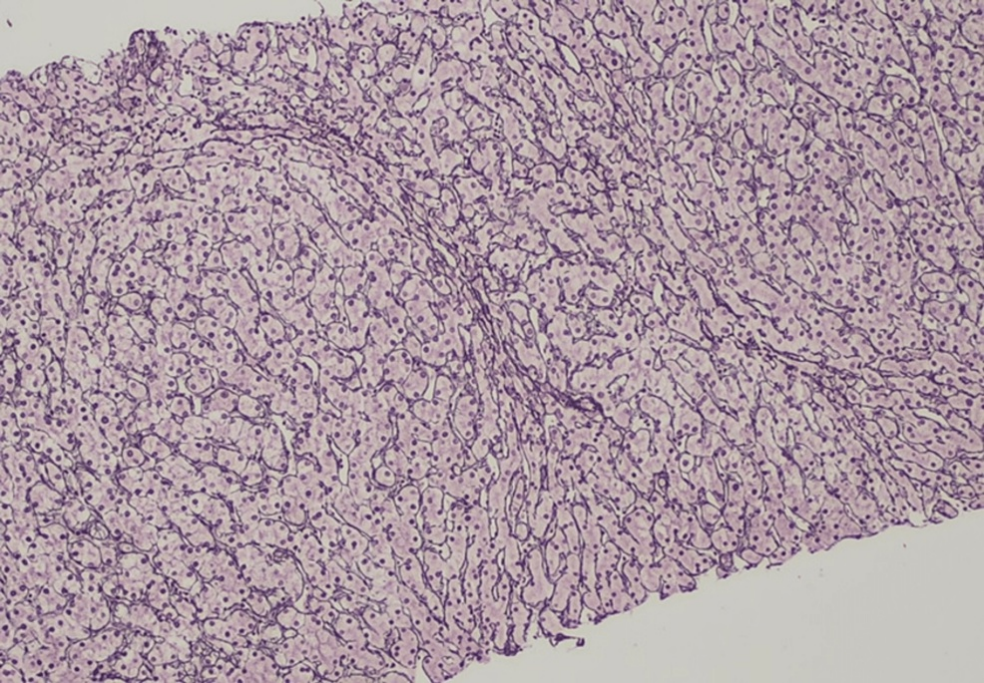
Liver biopsy, Gordon and Sweets' staining, 200x On histological evaluation, there were 27 portal spaces available with mild and focal lymphoplasmacytic inflammatory infiltrate, without injury to its constituents, and no interface activity. There was no fibrosis. The liver parenchyma exhibited nodulation on Gordon and Sweets' staining, with central-hypertrophied hepatocytes surrounded peripherally by atrophic hepatocytes, sometimes compressing portal spaces. The changes found translate nodular regenerative hyperplasia and disorders of efferent venous flow/low output compatible with non-cirrhotic portal hypertension.

Transcutaneous echo-guided liver biopsy revealed two fragments, with 2.2cm in total, without architectural changes. On histological evaluation, there were 27 portal spaces available with mild and focal lymphoplasmacytic inflammatory infiltrate, without injury to its constituents and no interface activity. There was no fibrosis. The liver parenchyma exhibited nodulation on Gordon and Sweets' staining, with central-hypertrophied hepatocytes surrounded peripherally by atrophic hepatocytes, sometimes compressing portal spaces (Figure 3). The changes found translate NRH and disorders of efferent venous flow/low output compatible with NCPH.

Treatment and follow-up

The dosage of spironolactone and furosemide was increased to 200mg and 40mg respectively, accompanied by paracentesis with ascites control. We started a gradual reduction of corticosteroid therapy, assuring continuous monitoring and control of the haemolytic anaemia. The patient was encouraged to perform physical exercise for muscle strengthening and a sodium-restricted diet (about 2g/dL) to help control ascites.

The patient presented no recurrence to date with a total follow-up time of four years, with consultations and abdominal ultrasound every six months and upper gastrointestinal endoscopy with no oesophageal varices or mucosa-associated lymphoid tissue lymphoma related to the CVID.

## Discussion

We presented the case of a 48-year-old woman with asthenia and ascites in the last three months, which led us to the diagnosis of INCPH. With a previous diagnosis of CVID, INCPH is a possible and often overlooked complication of CVID and, in this case, the cause of the severe ascites.

INCPH falls within the pre-sinusoidal intrahepatic causes of PH. It is more frequent on the Asian continent and may be associated with lower socioeconomic status, ethnicity, living conditions or exposure to different pathogens [9-11]. Nevertheless, the definition of idiopathic, chronic, or recurrent infections, exposure to drugs or toxins, HIV infection, immunological disorders, genetic predisposition, and hypercoagulability appear as predisposing factors [9-11].

Although the pathophysiology of the disease is unknown [9-11], an increase in splanchnic blood flow and portal venopathy (caused by thrombosis or obliteration secondary to autoimmune injury, endothelial injury, or hypercoagulability) can be proposed as a causative factor [9].

The predominant clinical presentation in these patients is PH and its subsequent complications, with haemorrhage due to rupture of oesophageal varices (that are present at diagnosis in 85-95% of patients) being the most common [9,11]. As liver function is normally preserved, the prognosis of bleeding by rupture of oesophageal varices is better than in cirrhotic patients [9,11]. Ascites is present in 50% of the patients and generally appears in the context of precipitating factors, mainly infections and rupture of oesophageal varices, with no reported cases (known to the authors) whose precipitating factor has been haemolytic anaemia associated with CVID [9,11].

Portal vein thrombosis is common, especially in patients with HIV infection, or who have previously had a bleeding episode. Hepatic encephalopathy is rare [9,11]. Manifestations of hypersplenism (anaemia, thrombocytopenia, and leukopenia) and symptoms of massive splenomegaly may also appear [9-11].

INCPH, an exclusion diagnosis, requires all the following criteria: (i) unequivocal clinical signs of PH; (ii) liver biopsy that excludes cirrhosis or fibrosis; (iii) proof of permeability of the hepatic and portal veins through imaging tests; and (iv) exclusion of hepatic and extrahepatic pathologies that develop with PH [9,11].

The diagnostic workup should include a meticulous medical history (exclude pathologies, medications, toxins or drugs associated with PH) and complementary diagnostic tests to assess the previously mentioned diagnostic criteria as done in our patient. Other tests may assist in the differential diagnosis of cirrhotic vs non-cirrhotic PH, namely percutaneous elastography and measurement of the hepatic venous pressure gradient [9,11].

Liver biopsy is extremely important for the diagnosis of INCPH since it allows for the exclusion of cirrhosis and other pathologies that cause PH [9-11]. Histological changes associated with INCPH are varied and none pathognomonic. They range from obliterative portal venopathy, portal triad hypoplasia, phlebosclerosis, and portal and hepatoportal sclerosis to sinusoidal dilation, presence of aberrant vessels, perisinusoidal fibrosis, and NRH [9-11]. In the liver biopsy of our patient, we found NRH. This histological abnormality results from intrahepatic vasculopathy and leads to hepatocyte damage and subsequent regeneration. The regenerative micronodules, associated with an autoimmune mechanism [1,7,9], will compress the entire surrounding area. In patients with CVID, NRH is most often manifested by elevation of alkaline phosphatase without elevation of bilirubin or PH, and it is associated with reduced survival compared to patients with NRH, but no CVID [1].

Our patient presented with manifestations of PH, having a preserved liver function. Unconjugated hyperbilirubinemia was present, most probably linked to the haemolytic anaemia, which even though under control, was still active. All diagnostic criteria of INCPH were met in this patient.

As previously mentioned, patients who present with ascites usually have a reason for the decompensation, the most common being rupture of oesophageal varices and infections. Despite not having any of the described causes of flare, our patient had a recent episode of severe haemolytic anaemia, for which she was still being treated with high-dose corticosteroids. Despite extensive research, we did not find any cases reporting ascites associated with episodes of haemolytic anaemia in patients with CVID. Nevertheless, we should consider it as a possible decompensation factor.

There is no curative treatment for INCPH or specific guidelines. Thus, therapy should be individualized and aimed at the prevention and treatment of PH complications, following the guidelines for patients with liver cirrhosis. Anticoagulant therapy may benefit patients with hypercoagulability [9-11].

The prognosis of patients with INCPH is better than that of those with liver cirrhosis, and screening for hepatocellular carcinoma is not recommended [9]. Factors of worse prognosis include the development of ascites, and the presence of immunological and neoplastic diseases, both present in our patient. Another factor to consider is NRH, which, as previously mentioned, confers decreased survival.

More recently, and with the advancement of knowledge about this pathology, a new entity called porto-sinusoidal vascular disease (PSVD) was proposed. This entity intends to describe a group of hepatic vascular diseases that course with damage to the sinusoids and hepatic venules, regardless of the presence or absence of PH [12,13]. Furthermore, it intends to include patients with other chronic liver diseases that are a priori excluded from the diagnosis of INCPH [12,13]. The diagnostic criteria for this new entity require a liver biopsy measuring 20mm or more with the presence of at least 10 portal tracts associated with one specific sign of PH or one histological lesion specific to PSVD. Alternatively, the diagnosis can be made in the presence of one nonspecific sign for PH and one nonspecific histological lesion for PSVD [12,13]. The patient has a liver biopsy that met the quality criteria and was identified NRH, a histological lesion specific for PSVD. Thus, the diagnosis of PSVD can be proposed for this patient at the expense of the diagnosis of INPCH. However, although we can assign a new name to this pathology, the approach and treatment of this entity do not change.

## Conclusions

INPCH should be considered in CVID patients with new onset of ascites after extensive evaluation and non-probable cause founded. This is a rare form of liver involvement of CVID and is an exclusion diagnosis. Liver biopsy is extremely important and none of the possible findings is pathognomonic of INPCH, including NRH (another possible cause for PH). Treatment should be individualized and aimed at the prevention and treatment of PH complications, following the guidelines for patients with liver cirrhosis. The prognosis of patients with INCPH is better than that of those with liver cirrhosis.
